# The Outcome of COVID-19 Lockdown on Changes in Body Mass Index and Lifestyle among Croatian Schoolchildren: A Cross-Sectional Study

**DOI:** 10.3390/nu13113788

**Published:** 2021-10-26

**Authors:** Gordana Kenđel Jovanović, Nataša Dragaš Zubalj, Sanja Klobučar Majanović, Dario Rahelić, Valentina Rahelić, Jelena Vučak Lončar, Sandra Pavičić Žeželj

**Affiliations:** 1 Department of Health Ecology, Teaching Institute of Public Health of Primorsko-Goranska County, 51000 Rijeka, Croatia; sandrapz@medri.uniri.hr; 2 Department of School and University Medicine, Teaching Institute of Public Health of Primorsko-Goranska County, 51000 Rijeka, Croatia; natasa.dragas-zubalj@zzjzpgz.hr; 3 Department of Endocrinology, Diabetes and Metabolic Diseases, Clinical Hospital Centre Rijeka, 51000 Rijeka, Croatia; sanja.klobucar@medri.uniri.hr; 4 Department of Internal Medicine, Faculty of Medicine, University of Rijeka, 51000 Rijeka, Croatia; 5 Vuk Vrhovac University Clinic for Diabetes, Endocrinology and Metabolic Diseases, Merkur University Hospital, 10000 Zagreb, Croatia; dario.rahelic@gmail.com; 6 School of Medicine, University of Zagreb, 10000 Zagreb, Croatia; 7 School of Medicine, Josip Juraj Strossmayer University of Osijek, 31000 Osijek, Croatia; 8 Department of Nutrition and Dietetics, University Hospital Centre Zagreb, 10000 Zagreb, Croatia; valentina.rahelic@kbc-zagreb.hr; 9 Department of Health Studies, University of Zadar, 23000 Zadar, Croatia; jelenaby23@yahoo.com; 10 Department of Health Ecology, Faculty of Medicine, University of Rijeka, 51000 Rijeka, Croatia

**Keywords:** children, COVID-19 pandemic, dietary habits, lifestyle, obesity, physical activity, screen time

## Abstract

Globally, the COVID-19 pandemic altered adults’ and children’s lifestyles and habits, causing an increase in body weight. Adolescents are sensitive to habit changes and, because of their insufficient capacity to deal with the unexpected COVID-19 changes, were at greater risk of noncommunicable disease development due to the consequences of adopting unhealthy habits. The survey aimed to reveal the changes in nutritional status and lifestyle habits of school children in Croatia and to assess their nutrition knowledge and emotional state and feelings about COVID-19 lockdown. Self-reported data from 1370 school children aged 10 to 15 years were obtained to examine the influence of the lockdown on their nutritional status, lifestyle and emotional status, and to assess their nutrition knowledge. The study revealed that the COVID-19 lockdown has caused an increase in the proportion of overweight and obesity among Croatian school children who changed their lifestyle habits towards being less physically active, spending more time using screen-based media and revealing potential psychological distress. However, the schoolchildren had a high adherence to the Mediterranean diet assessed with the Mediterranean Diet Quality Index for children and adolescents (KIDMED) index and had good nutrition knowledge. Public health programs promoting a healthy lifestyle and involving the whole family, in a school environment, could provide children with a healthy adulthood.

## 1. Introduction

The coronavirus disease 2019 (COVID-19) [1] has caused a worldwide pandemic recently, where control and prevention measures led to outdoor activity restrictions, social distancing and the closedown of public facilities, including public schools. In Croatia, public schools started with full online teaching at the beginning of March 2020 [2] which was eased after two months only for the youngest children, i.e., those from first to fourth grade of elementary schools who returned to school, while older children remained on full online teaching until the end of the school year in June. In September 2020, all elementary school children in Primorsko-Goranska County in Croatia started their school year by attending school, but those from the fifth grade and older stayed at home on full online teaching in October and remained that way, with short returns to school, until the beginning of May 2021, when they all returned to school and stayed until the end of the school year in June 2021 [2,3]. The period of staying at home because of the COVID-19 lockdown measures may have caused children’s usual lifestyle and school habits to change. Adolescence is an important human development period, which includes psychological, social and lifestyle changes. Adolescents are particularly sensitive to changes in their newly acquired freedom in lifestyle habits and may have insufficient capacity for coping with unexpected changes due to COVID-19 lockdown [4]. Health-related lifestyle habits implemented in early life are mostly maintained in adulthood, and, since they are significant determinants of health status in adulthood [5], it is important to observe children’s habits, especially if there is a possibility of their change.

Due to COVID-19 lockdown measures, decreased physical activity (PA) and unhealthy dietary patterns were shown to be the key factors mediating the negative influence on health [6,7,8,9,10,11,12,13,14,15,16,17,18,19,20,21]. Recent studies from all over the world examined the COVID-19 lockdown influence on elementary schoolchildren’s health behavior changes and perceived reduced overall physical activity [6,7,8,9,10,11,12,13,14,15,16,17]. Yomoda and Kurita [14], in their recent review of studies conducted in the first half of 2020, observed that the decrease of PA was greater in boys and older children, although one study noticed the overall PA increase due to the increase in children’s habitual physical activities [8,14]. The authors suggested that children’s PA could remain the same or be increased by parental support and engagement, making children’s available time more effective, also arranging locations and forms of physical activity [14]. Jurak et al. [12] emphasized that only 2 months of self-isolation due to the COVID-19 lockdown erased over 10 years of hard-fought health achievements attained from national public health policies and PA interventions.

Recent studies regarding changes in eating habits during the COVID-19 lockdown conducted among adolescents showed an increased intake of non-nutritious foods [6,7,21] but also an increased intake of fruits and vegetables [11,20], dairy products [17], or increased consumption of all food groups [21]. It was also shown that there was no significant change in food group consumption [17,19] or dietary pattern [18] when compared to the time before the lockdown.

The change of lifestyle due to COVID-19 lockdown measures may have resulted in body weight increase because of the increase of obesogenic factors such as physical inactivity, increased sedentary behavior including increased screen watching time, and practicing an unhealthy diet, such as frequent snacking and ultraprocessed food intake [21]. A recent review noted an increase in body weight of 0.5 to 1.8 kg among adults [22], while studies conducted among children have recorded a similar increase in body weight [16,21], where parents noted increased body weight in a quarter [18] and a third [11] of their children.

It was noticed that the COVID-19 lockdown has caused a significant, substantial increase in various psychological distresses, such as depression, anxiety, and stress among schoolchildren, which was pronounced among older adolescents and in vulnerable children [23]. Additionally, the COVID-19 lockdown revealed that the problematic use of gaming, social media, and smartphones among Chinese primary school children showed mediating effects on the association between psychological distress and screen time use [24]. The adverse psychological reactions to the COVID-19 pandemic such as anxiety, depression, boredom, fear, stress together with family financial loss and social media information overload caused the changes in lifestyle habits and weight gain in adults [22], and possibly among children, which should be further investigated.

Recent studies in adults [22], children and adolescents [11,16,17,18,21,25] showed that the COVID-19 pandemic resulted in a changed lifestyle which was associated with weight gain that could lead to obesity that is associated with metabolic changes that increase the risk of noncommunicable diseases such as diabetes and cardiovascular disease [26]. It could be assumed that the lockdown could give rise to worldwide obesity rates [27]. To emphasize the health-related connection between obesity and COVID-19 and a noticeable aggravation in obesity rates due to the lockdown measures, the new term “covibesity” was formed [26]. During the COVID-19 lockdown in Croatia, adults with a body mass index (BMI) above 25 kg/m^2^ had a greater probability of weight gain, while those with a BMI less than 25 kg/m^2^ were more prone to increase their PA and were more adherent to the Mediterranean diet (MD) [28]. Croatian adolescents significantly reduced their PA level, mostly those living in urban areas [29]. A fifth of surveyed Croatian students reported weight gain [30], their diet did not significantly change when compared to adherence to the MD, but they increased adherence to the MD pyramid for fruit, legumes, fish, and sweets, and decreased for cereals, nuts, and dairy intake during the COVID-19 lockdown.

In 2009 and 2015 in Croatia, the overweight and obesity prevalence in children aged 11 to 15 years was around 18%, and it was higher among boys [31,32]. The change in the overweight and obesity prevalence among Croatian schoolchildren during the COVID-19 pandemic has not yet been assessed. It could be hypothesized that the COVID-19 lockdown may have increased the prevalence of overweight and obesity, therefore this study aimed to investigate the change in BMI and lifestyle habits of schoolchildren aged 10 to 15 years in Primorsko-Goranska County, Croatia, also to assess their nutrition knowledge, as well as their emotional state and feelings about the COVID-19 lockdown.

## 2. Materials and Methods

### 2.1. Study Design and Sample

This is a quantitative cross-sectional study among elementary school children of 5th to 8th grade (aged 10 to 15 years, 639 boys and 731 girls) in Primorsko-Goranska County, Croatia, based on an anonymous electronic survey, also known as an e-survey or web survey. Since this research was voluntary and anonymous, where no schoolchildren’s personal data were collected, their parents were not asked to sign permits to participate but were allowed to refuse to participate by signing a statement of nonparticipation after the research presentation. The research aims were presented to all the 59 elementary schools in Primorsko-Goranska County in Croatia. The consent to participate in this research was given by 15 schools (25%), 10 schools from urban areas and 5 schools from rural areas. The research protocol was explained to the school, physical education teachers, school children and parents who agreed to participate. By completing the online questionnaire, the children gave their consent to participate in the research where it was explained to them that their questionnaires were anonymous and does not reveal their identity. The inclusion criteria for this survey were students of both genders attending 5th to 8th grade in an elementary school from Primorsko-Goranska County, Croatia. The exclusion criteria were all elementary school children from 1st to 4th grade and those who did not entirely fill in the survey questionnaire. The calculated representative number of participants for this survey was 370, based on a 5% error threshold and a test power of 0.95 for the population size of 9662 schoolchildren, which was the number of school children attending 5th to 8th grade of elementary schools in Primorsko-Goranska County, Croatia, in the school year 2020/2021. The questionnaire was completed voluntarily by 3152 children (32.6% of all school children), but it was entirely completed by 1370 children aged 10 to 15 years (14.2% of all and 43.5% of children surveyed), which is almost four times larger than the calculated sample size. Data from the fully completed questionnaires of 1370 children were further analyzed and presented in this study.

### 2.2. Body Weight, Height, and Body Mass Index

For children who agreed to participate in the research, their physical education teachers provided data of his/her measured body weight and height which were measured when children went to school in September 2020 at the beginning of the school year, and in May 2021. Body mass index (BMI) was calculated as a ratio of weight to height (kg/m^2^). Centile grids for BMI according to age, gender, and height from “Croatian Anthropometric Reference Values for School Children and Youth” [33] were used to define participants’ underweight, normal weight, overweight, and obesity.

### 2.3. The Questionnaire

The questionnaire consisted of four sections: socio-demographic data; children’s lifestyle habits which included physical activity (sitting, sports, activities during leisure time), sleeping (hours/day), screen time use (TV/PC/tablet/mobile phone) and dietary habits; nutrition knowledge questions; and questions about their emotions and feelings considering COVID-19 lockdown. Sociodemographic data included information about their age, gender, grade and school, and body weight and height measurements received from their physical education teacher. The lifestyle habit questions referred to the time when they went to school (before COVID-19 lockdown), and to the time when they were in lockdown (during COVID-19 lockdown). The questions were the same in each time section. The questions were about their habits of physical activities (organized and nonorganized), sitting time, screen time use, and sleeping time, in which schoolchildren recorded time doing them, frequencies offered as one to two; three to four, five to six times per week, and every day. They noted time quantity doing those activities offered as less than half an hour; half an hour to one hour; one to two; three to four, five to eight hours, and more than eight hours. To calculate their average total weekly PA (MET-min/week) the products of noted time for each item by a metabolic equivalent tasks (MET) value that was specific to each category of PA (vigorous PA = 8.0 METs, moderate PA = 4.0 METs, walking = 3.3 METs, sitting = 2.5 METs and sleeping = 0.95 METs) were added. According to provided sum, we scored participants according to their PA level as low (<600 MET-min/week), moderate (600–3000 MET-min/week), and high (>3000 MET-min/week). Dietary habits contained questions about breakfast eating frequencies (less than once; one to three; four to six times per week, and every day) and composition (dairy, wholegrain cereal products, bakery products), and 21 questions about food intake (fruits, vegetables, legumes, cereals, dairy, meat, fish, nuts, olive oil, fast food, sweets and desserts, sweetened beverages), which they noted in offered frequencies (less than once; once; two to three; four to six times a week; every day once; every day two or more times) and in offered quantities (less than medium, medium, larger than medium). Given that Croatia as a Mediterranean country encourages the Mediterranean diet as a reference dietary pattern [34], and part of Croatia where this survey was conducted resides by the Mediterranean Sea where the traditional Mediterranean diet is the inherited diet, the diet quality of school children was assessed with the Mediterranean Diet Quality Index for children and adolescents (KIDMED) index to grade the adherence to the Mediterranean diet (MD) [35]. The KIDMED score ranged from 0 to 12. The consumption of fruits, vegetables, legumes, wholegrain cereals, nuts, milk and dairy products, yogurt, and olive oil was signified as a positive aspect concerning MD and was assigned with a value of +1, while the consumption of baked goods, sweets and desserts, fast food, and skipping breakfast was considered as a negative aspect and was assigned with a value of −1. The adherence to MD level was divided according to the provided KIDMED overall score into low adherence to MD (≤3 points), medium adherence (4 to 7 points), and high adherence to MD (≥8 points). The assessment of nutrition knowledge (NK) considered the scoring of 14 questions based on nutrition lessons in the elementary school biology curriculum. The NK score also included their opinion about what they considered as a healthy breakfast and healthy meal and about physical activity. Based on the overall NK score, participants were distributed into low NK (≤4 points), moderate (5 to 7 points), and high NK (≥8 points). The questionnaire included five questions about their feelings and worries about COVID-19 lockdown staying at home as follows: “I feel lonelier.”, “I feel happier.”, “I miss my school friends.”, “I feel more successful in my learning.”, “I’m afraid to go back to school, I rather stay at home.”. Those questions were scored by the participants with the 5-point Likert scale as follows: 1 = no, not at all; 3 = neither yes, neither no; 5 = definitely yes. All received questionnaires were checked at the survey center for completeness, and those questionnaires with missing questions were excluded.

### 2.4. Statistical Analysis

All provided data were first tested for normality of distribution with the Kolmogorov−Smirnov test. The numerical data were presented as mean ± standard deviation and tested with an independent Student *t*-test for differences according to gender. A dependent *t*-test was performed for repeated measures regarding the time before online and during online teaching among boys and among girls. The ordinal data of grouping variables are presented as number and proportions and tested with χ2–test according to gender, physical activity and BMI-for-age level. The McNemar’s test was performed for the determination of differences in dichotomous dependent variables between boys and between girls. The odds ratio and 95% confidence intervals were calculated in the univariate logistic regression to test the change in overweight and obesity with the change of monitored variables. Statistical significance was set at *p* < 0.05. All tests were done with Statistica 12.7 for Windows (Statsoft Inc, Tulsa, OK, USA).

## 3. Results

### 3.1. Participants’ Characteristics

The study sample consisted of 1370 participants, with statistically more girls than boys (53.4% and 46.6%; *p* = 0.013, respectively). Their mean age was 12.72 ± 1.17 years (boys 12.83 ± 1.18 years, girls 12.61 ± 1.15 years; *p* = 0.692). Regarding age categories, there were significantly the most participants (*p* = 0.049) in the age group of 12 to 13 years (51.9%, 318 boys, 393 girls), and more in the age group of 14 to 15 years (30.1%, 218 boys, 195 girls) than in the age group of 10 to 11 years (18.0%, 103 boys, 143 girls). There were statistically more participants residing in urban (71.7%) than in rural areas (28.3%) (*p* < 0.001), but with no difference according to gender (urban: 446 boys, 536 girls vs. rural: 193 boys, 195 girls; *p* = 0.148). Before COVID-19 lockdown, girls had significantly lower BMI values than boys (19.98 kg/m^2^, 20.53 kg/m^2^, respectively; *p* = 0.010). During lockdown there was a significant increase in BMI values in a total sample of participants (ΔBMI = 0.66 ± 4.39 kg/m^2^; *p* = 0.041), and by the same difference in boys (ΔBMI = 0.69 ± 6.27 kg/m^2^; *p* = 0.029) and in girls (ΔBMI = 0.62 ± 1.37 kg/m^2^; *p* = 0.002) (Table 1). The participants’ distribution regarding BMI-for-age level did not differ regarding gender before (*p* = 0.344) and during lockdown (*p* = 0.607), but there was a significant increase in overweight and obesity in boys (Δ2.0% (95% CI 0.9–3.1), 0.9% (95% CI 0.2–1.7), respectively; *p* = 0.007). In girls, there was a significant increase in overweight for 3.3% (95% CI 2.1–4.9), and a decrease in underweight for 4.1% (95% CI 2.8–5.8) (*p* = 0.001) (Table 1).

### 3.2. Physical Activity, Screen Time Use and Sleeping Habits

Before the COVID-19 lockdown, boys were significantly more active than girls (*p* < 0.001), who were in a significantly higher proportion having a low PA level than boys (22.0%, 16.3%, respectively; *p* = 0.003) (Table 1). Boys were significantly more engaged weekly in organized (*p* < 0.001) and in nonorganized sports activities than girls (*p* = 0.005). Most of the participants reported ≥ 7 h/day for class and homework sitting, girls more than boys (58.7%, 66.6%, respectively; *p* = 0.002). Two-thirds of the participants reported times of more than 2 h per week for using PC/tablet/mobile phone, and more than 90% of them watched TV less than 2 h per day, with no significant difference regarding gender. Average sleeping time was around 8 h per day, but there were many who reported a sleeping ≤ 7 h/day, and a third of them slept ≥ 9 h/day, with no difference regarding gender (Table 1). During COVID-19 lockdown, both boys and girls significantly reduced their average PA (Δ1468.9 ± 1107.8 MET-min/week; *p* < 0.001, Δ1234.1 ± 964.8 MET-min/week; *p* < 0.001, respectively), and both had a significant increase in those with low PA level (Δ59.5% (95% CI 55.6–63.3); *p* < 0.001, Δ53.4% (95% CI 49.7–57.0); *p* < 0.001, respectively) (Table 1). Boys were significantly more engaged in organized sports activities that were organized ≥ 4 times per week than girls (*p* < 0.001), who were significantly engaged 2–3 days in a week in organized sports, while in the time before lockdown it was for 14.6% more (*p* < 0.001). Both boys and girls significantly reduced the number of days in a week for doing their nonorganized sports activities (*p* = 0.007, *p* < 0.001, respectively). Participants raised their time for class and homework sitting (Δ5.3% (95% CI 4.2–6.7); *p* = 0.008), boys significantly more (Δ13.1% (95% CI 10.6–16.0); *p* < 0.001), and girls without significant difference (Δ−1.5% (95% CI 0.8–2.7); *p* = 0.544). Both boys and girls significantly raised their time in a day for PC/tablet/mobile phone use (Δ10.2% (95% CI 7.9–12.8); *p* < 0.001, Δ12.2% (95% CI 9.9–14.8); *p* < 0.001, respectively). Both groups also significantly increased their daily time for watching TV (Δ5.2% (95% CI 3.6–7.2); *p* = 0.003, Δ6.6% (95% CI 4.9–8.6); *p* < 0.001, respectively). Although, on average, most of the participants slept during lockdown ≤ 7 h/day, there was a significant increase of those who slept between 8 to 9 h/day (boys Δ3.9% (95% CI 2.6–5.7); *p* = 0.001, girls Δ2.1% (95% CI 1.2–3.4); *p* = 0.044) (Table 1).

### 3.3. Dietary Habits and Nutrition Knowledge

Participants’ assessed dietary habits with the KIDMED score showed that, on average, their diet highly adhered to the MD, and a significantly high share of participants had a diet that moderately and highly adhered to MD (*p* < 0.001). There was no significant difference according to gender (Table 2).

During the lockdown, the increase of school children in the low-activity group and the decrease in moderate- and high-activity groups were noticed. Therefore, the school children were further divided into two groups: a low-activity group (1035 participants), and an activity group (335 participants) that included those who were moderately and highly active. All their compliances with 16 KIDMED items are presented in Table 3 together with the compliances of boys and girls, and BMI-for-age classes. It was noticed that there was no statistically significant difference according to activity classification in all KIDMED items, but those who were active had slightly better compliance with all KIDMED items than those who had low activity. Almost a third of schoolchildren (26.9%) did not consume a fruit every day, but a second fruit per day was consumed by half of the schoolchildren (53.4%). Although all school children consumed one meal of vegetables (100.0%), only 53.4% of them had two meals of vegetables a day, girls, physically active children and statistically significantly those with obesity (*p* = 0.038) more than other groups (Table 3). Although, on average, only a third of school children consumed fish two or more times a week, boys consumed statistically significantly more (*p* = 0.032), as well as legumes (*p* = 0.002), grain and whole-grain products (*p* = 0.037), yogurt or cheese every day (*p* = 0.012), whole grains, milk and milk and dairy products for breakfast (*p* = 0.047) than girls. Boys statistically consumed fewer sweets or salty snacks in a day (*p* = 0.002) and skipped breakfast less (*p* = 0.001) than girls (Table 3). Girls statistically more than boys reported using olive oil for cooking (*p* = 0.047) and eating fast food less than once per week (*p* = 0.010). Around half of all schoolchildren stated they did not consume baked goods or pastries for breakfast (54.9%), those with obesity statistically the least (*p* = 0.033), and they also reported consuming fewer sweets or salty snacks in a day than the other BMI groups (*p* = 0.019).

The nutrition knowledge of the surveyed schoolchildren was on average high, and statistically, girls scored better than boys (9.2, 8.8; *p* = 0.001, respectively) (Table 2). There were statistically more of those in the high knowledge group (68.7%; *p* = 0.007) than in the moderate or low group (28.3%, 21.0%, respectively). The detailed items of the nutrition knowledge questionnaire and the share of schoolchildren that correctly answered the nutrition knowledge questions according to gender are presented in Supplementary Table S1.

### 3.4. Factors Contributing to Overweight and Obesity before and after COVID-19 Lockdown

Before the COVID-19 lockdown, there were significantly lower odds for overweight and obesity in the older than among the younger participants (OR = 0.58; *p* = 0.004) which stayed significant and the same during lockdown (OR = 0.51; *p* = 0.001) (Table 4). The odds for overweight and obesity before the lockdown was significantly lower for those with moderate PA compared to those with low PA (OR = 0.71; *p* = 0.016). Those who were engaged in organized sports two to three times per week had lower odds for overweight and obesity than those engaged less than 2 d/week (OR = 0.59; *p* = 0.001), but reduced during the lockdown and stayed significant (OR = 0.23; *p* < 0.001). Similarly, those who were engaged more days in a week doing nonorganized sports activities had significantly lower chances for overweight and obesity (OR = 0.58; *p* = 0.002, OR = 0.63; *p* = 0.004) than those doing them less than 2 d/week. During the lockdown, the odds for overweight and obesity regarding engagement in organized sports more than 2 d/week rose almost twice (OR = 0.23, OR = 0.36; both *p* < 0.001) compared with those who were engaging in sport less than 2 d/week. Those who slept ≥ 9 h/day had significantly lower chances for overweight and obesity (OR = 0.75; *p* = 0.021) compared to those who slept ≤ 7 h/day. Class and homework sitting and screen use time were not significantly associated before and during lockdown (Table 4). During the lockdown, those who ate breakfast 4–6 times in a week or every day had lower odds for overweight and obesity during lockdown than breakfast skippers (OR = 0.46; *p* = 0.001, OR = 0.63; *p* = 0.019, respectively) (Table 5). Those who had more than one fruit in a day had statistical 4.42 more odds for overweight and obesity than those who did not eat any fruit (OR = 4.42; *p* < 0.001) (Table 5). Those who had two or more vegetable meals in a day also had 2.04 more odds for overweight and obesity than those who had one vegetable meal (OR = 2.04 *p* < 0.001). Those who drank less than one sweetened beverage in a day had statistically higher odds for overweight and obesity than those who drank it once or more in a day (OR = 8.64; *p* < 0.001). Similarly, those who ate sweet or salty snacks once a day had higher odds for overweight and obesity (OR = 1.48; *p* = 0.001) than those who ate those snacks frequently in a day (Table 5). Compared to the low nutrition knowledge group, those who had moderate nutrition knowledge had statistical higher odds for overweight and obesity (OR = 12.95; *p* < 0.001), likewise those with high knowledge (OR = 8.69; *p* < 0.001) (Table 5).

### 3.5. Psychological and Emotional Factors Regarding COVID-19 Lockdown

**Figure 1:**
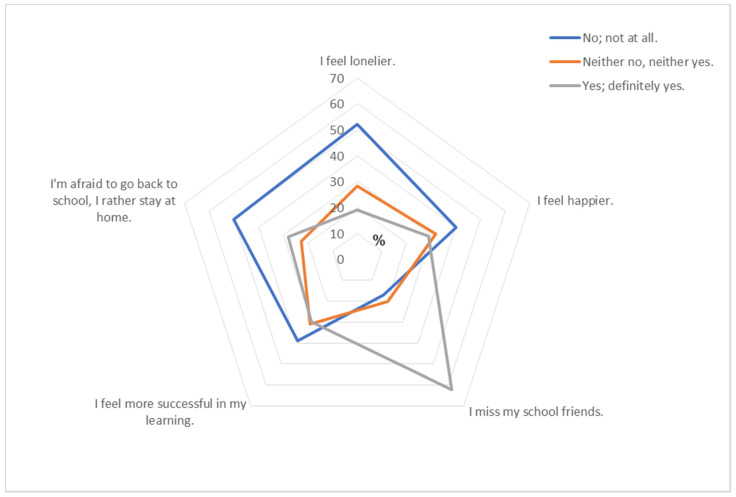
Graphical presentation of school children’ feeling and worries about COVID-19 lockdown and staying at home.

In Figure 1, feelings and worries related to COVID-19 lockdown are presented. On average, one-fifth of all schoolchildren felt lonelier during lockdown (19.5%), but there were statistically more of those who said no and not at all (52.2%, *p* < 0.001). There were statistically more of those who did not feel happy about the lockdown and staying at home than those who did (39.6%, 28.8%; *p* < 0.001, respectively). There were statistically more of those who missed their school friends than those who did not (62.5%, 17.4%; *p* < 0.001, respectively). The majority of schoolchildren did not feel successful in their learning during the lockdown (38.8%; *p* < 0.001), but there were almost equal numbers of those who did feel successful and of those with no opinion about that (30.4%, 30.8%, respectively). Although most of all schoolchildren were not afraid to go back to school and would not rather stay at home (49.9%, *p* < 0.001), still there was almost a third of those who were afraid and would rather stay at home (27.6%), more girls than boys (29.8%, 25.0%; *p* = 0.048, respectively). Details of this issue are presented in Supplementary Table S2.

## 4. Discussion

To the best of our knowledge, this is the first study on the nutritional status and lifestyle changes during the COVID-19 lockdown among Croatian schoolchildren from elementary schools in Primorsko-Goranska County aged 10 to 15 years, which includes their dietary habits, nutritional knowledge and psychological and emotional state concerning lockdown. In this cross-sectional study, in relation to before the COVID-19 lockdown, a significant increase in BMI, an increase in the proportion of boys with overweight and obesity and of girls with overweight was noticed. The prevalence of overweight and obesity among surveyed schoolchildren before the COVID-19 lockdown was 21%, which is lower than 23%, which was the prevalence of overweight and obesity assessed in 2019 among 6701 children of primary school in Primorsko-Goranska County, Croatia [36]. During the lockdown, the overweight and obesity prevalence among surveyed schoolchildren increased to 24%. The study results are in line with the observed weight gain in schoolchildren of similar age from Greece, Spain, Jordan and Palestine [11,16,18,21]. Such short-term increases in body mass in children, as observed in this study, have been shown to be linked to obesity in later life, and if obesity remains untreated in adulthood, it may cause disability and shorten life span, because obesity is associated with chronic low-grade systemic inflammation, which has been acknowledged as one of the major drivers of chronic health conditions such as cardiovascular disease and type 2 diabetes mellitus as major obesity-related causes of death, and other conditions associated with chronic inflammation such as metabolic syndrome, non-alcoholic fatty liver disease, hypertension, and hyperlipidemia [25,26,27,37,38]. The association of changed lifestyle habits during the COVID-19 lockdown with the incidence of overweight and obesity showed that the older teens had significantly higher odds for overweight and obesity compared to the youngest, those who less often had organized activities during the week, those who were more often physically active during the week doing nonorganized activities, those who ate breakfast four to six times a week, those who ate more fruit and vegetable meals in a day, and those who ate fewer sweet and/or salty snacks and drank fewer sweetened beverages. Dietary habits should be further examined since the study results showed that healthy habits were associated with overweight and obesity, but this could be due to the observed increase in the prevalence of overweight and obesity.

Sedentary lifestyle habits were shown to be associated with a higher risk of obesity and cardiometabolic disease both in adults and children [39], therefore, the observed increase in overweight and obesity prevalence among surveyed children can aggravate the possible risk of obesity-related comorbidities in adulthood. There are well-known PA-associated health benefits for adolescents, such as cardio-metabolic properties, motor skill development, bone density, psychological health and emotional regulation [40]. The study showed a significant decline in PA level during the lockdown, where most of the schoolchildren, more boys, lowered their overall physical activity level, and especially being less engaged in organized sports activities. There was a significant decrease in the number of days in a week doing nonorganized activities, which was higher among girls. All schoolchildren significantly increased their time for overall sitting, including sitting at class and for homework, which was higher in boys than in girls, because of the use of digital devices for online teaching and homework, likewise spending more of their time at home. The observed results were comparable to the perceived PA results from similar studies done among schoolchildren during the COVID-19 lockdown [6,7,8,9,10,11,12,13,14,15,16,17].

The use of a PC/tablet/mobile phone, watching television and playing video games, known as “screen-based media use behavior”, increase sedentary habits and therefore, are associated with various negative health consequences [41]. It is recommended that children aged 5 to 17 years have ≤2 h of recreational screen time a day [42]. Surveyed schoolchildren during lockdown significantly raised their time for using their PC/tablet/mobile phone and for watching TV, more girls than boys, as expected, because of the use of digital devices for online teaching and homework and spending more time at home. The increase in time for screen-based media observed in our study was also observed in similar studies across the world [6,7,8,10,11,15,16,17,21]. Excessive and inappropriate use of screen-based media may affect children’s development and health, because of the greater risk of health complications and mental health issues that has been noted, and the negative effects of cognitive, linguistic, social, and emotional development [43], so it is necessary to monitor and educate adolescents about proper and adequate screen-use time in the future. The observed increase in screen-based media use by the surveyed schoolchildren could cause a delay in bedtime and shortened sleep duration as a consequence. Not enough sleep in schoolchildren may influence their emotional health, attention span, immune function and cardiovascular health [44], which all may have affected mental health interference during the COVID-19 pandemic [45]. The sleeping habits of surveyed schoolchildren also changed during the lockdown, they slept little more than before the lockdown, boys more than girls, and surveyed schoolchildren slept on average for a shorter time than schoolchildren in a Croatian survey in 2015 [32]. These minor changes in sleep duration were also observed in recent similar studies among schoolchildren [6,9,10,11,18,20,21]. Perceived minor sleep changes among surveyed schoolchildren can be attributed to the fact that their parents wake them up for online classes, so the sleep change is not significant as it was among Croatian students [30].

Since this study was conducted at the end of the school year, most of which was under lockdown measures (more than 6 months), when teaching was conducted online and school children were at home, so no recollection of eating habits related to the period before the lockdown was done, due to possible recall errors that would affect the correctness of the data on dietary habits. The study questionnaire was administered at the beginning of return to school, so schoolchildren noted their dietary habits related to the time during the lockdown. The importance of healthy eating and food choices during adolescence is well known for supporting their growth and likewise preventing health problems in adulthood [46]. A current perspective on nutritional recommendations during the COVID-19 lockdown promotes the nutrients, foods, and dietary patterns [47] that are all included in the MD, which is recommended for the prevention and management of cardiovascular disease, diabetes type 2, obesity and other inflammatory-related conditions, based on which, the MD is considered an anti-inflammatory diet [38,48]. The assessed dietary habits of the surveyed schoolchildren with KIDMED score showed a high share of those with a diet that adhered to the Mediterranean diet. Similar was noticed among Greeks [11], and Spaniards [18]. The available data of the changed dietary habits of school children during COVID-19 lockdown are mixed and limited. Some of them presented a higher consumption of fruits and vegetables [6,7,11,20], lower consumption of fast food and energy drinks [11,20], higher consumption of all food groups [16], and non-nutritional foods [21]. This research showed that overweight and obese school children consumed less fruit, fish, grains and whole grains, nuts, dairy products, sweets and candies, used less olive oil for cooking, and consumed more vegetables, legumes and fast foods than those with underweight and normal weight. Although the girls more frequently skipped breakfast and consumed sweets more often, they had healthier dietary habits when compared to the boys. This research also noticed that there were no significant differences in the assessed dietary habits regarding activity level, although those who had low activity had unhealthier dietary habits, such as eating less fruit, vegetables, nuts, legumes, dairy and eating more fast food, sweets, and a more frequently skipping breakfast than those who were more active. The reason for these results may be that during the lockdown, those schoolchildren with healthy eating habits also reduced their level of physical activity, as they retained them during the lockdown, which could be why there was no significant difference found when comparing them to those who were more physically active. Retaining dietary habits during the lockdown period could also explain why those children with overweight and obesity also had healthier dietary habits when compared to children with normal weight. Further research is needed to explore the causal association of physical activity and dietary habits with overweight and obesity among schoolchildren during the COVID-19 lockdown. The Croatian study of adolescents and students observed no significant change in dietary habits with adherence to MD during the lockdown [30], but their diet seems to be moving away from the MD, as was also observed a few years ago among younger adults in the same area of Croatia as in this study [49]. Observed high adherence to MD in this study could be due to their staying at home and having more cooked meals, and the availability of fruits, vegetables, dairy products, and nuts. There is a possibility that fewer snacks, fast food and bakery products were bought, i.e., that parents took more care of their own and their diet and cooked their meals at home, because they probably stayed at home more because of their more frequent work from home. A Croatian study among adults during lockdown showed [28] that more than half of them increased their cooking frequency and their consumption of vegetables, legumes, fish and seafood, which explains our study results of high adherence to a MD. This study’s results are of great importance since the observed healthy dietary habits among the school children should remain in the future. It is important to further explore the dietary habits of school children and their parents in the future when they are not spending so much time at home as they were during the lockdown.

Recently, it was reviewed that higher nutrition knowledge among children and adolescents is mostly associated with normal weight [50], but there were also associations between higher nutrition knowledge and higher body weight but healthier food choices [51], supposing that having good knowledge about nutrition, children and adolescents still do not follow healthy eating habits, which is often under the influence of peers, skills and choices. The assessed nutrition knowledge in this study showed that a significantly high proportion of surveyed schoolchildren had moderate to high nutrition knowledge. Because of the small proportion of those with low nutrition knowledge, it was noticed that significantly higher odds for overweight and obesity were found for those who had moderate and high nutrition knowledge compared to those with a low one, but those with moderate nutrition knowledge had 1.49 times higher odds for overweight and obesity than those with high nutrition knowledge, which means that those with lower knowledge about nutrition had greater odds for overweight and obesity. Other associations of overall nutrition knowledge and its items will not be further studied in this research.

As it was supposed that COVID-19 lockdown could affect schoolchildren’s mental health due to social distancing, physical isolation from their friends, peers, teachers, extended family, and community, it was shown that the perceptions of poorer mental health among adolescents were pronounced in recent but limited studies across the world [52,53], more among older adolescents [53], and girls than boys [53]. In this study, most of the schoolchildren were undecided about the feeling of being lonelier during the lockdown, and a fifth of them felt lonelier. In addition to that, there were more of those who did not feel happier about their stay at home. Most of the surveyed schoolchildren missed their school friends. It was shown that the presence of psychosocial stressors such as social isolation during childhood and adolescence is significantly correlated with a risk factor for depression and with higher inflammation later in adulthood [54,55], which certainly indicates a need for further exploration among schoolchildren. Although most of the surveyed schoolchildren did not feel more successful in their learning during the lockdown, there were equally those who had no opinion and those who felt more successful. Half of them did not feel afraid to go back to school, but a third stated they were afraid of returning to school and would rather stay home. Croatian lockdown measures were not as strict as they were in some other parts of the world, the status and degree of intensity and duration of COVID-19 restrictions differed between countries and regions; in Croatia, it was allowed to go outside for recreation, but with social distancing. Still, the observed psychological distress, including fear, among schoolchildren could have an influence on their future mental health and worsen the atmosphere of fear, which could lead to growth of maladaptive anxiety [56], and this should be taken into account and be investigated in the future.

This study on changes in nutritional status and lifestyle habits among schoolchildren during the COVID-19 lockdown showed a significant increase in the prevalence of overweight and obesity, screen-based media use, a significant decrease in PA level where most of the children had a diet that highly adhered to the MD and with good nutrition knowledge, with a presence of psychologically negative concerns about COVID-19 lockdown. All of these could be considered as the study strengths, along with the fact that the measures of body weight and height were done by the same person and under the same conditions, before and a short time after the lockdown, which makes the data of nutritional status more accurate. Those data represent a significant contribution to the scientific society, bringing a snapshot of the Croatian school children’s lifestyle habits change during the COVID-19 lockdown upfront, which should certainly be monitored in the future. However, the observed results of this cross-sectional study were gathered from the self-reported responses of 1370 schoolchildren, which were collected from the web-based questionnaire. Those could be regarded as study limitations, and therefore should be considered with caution in the conclusions, because of the study nature, the number of participants and their possible bias in self-reporting data, although, during the lockdown period, this method of survey represented a unique alternative approach for data collection. Another limitation of this study could be that it presents the results of a third of schoolchildren attending primary schools in the Primorsko-Goranska County and that less than half of the children who entered this survey completed the entire questionnaire. This, however, could be considered a study strength because this study showed the results of those children who had completely filled in the questionnaire. A further study limitation is that no causal relationship was studied between obesity and changes in physical activity habits, while the change in dietary habits was not assessed because schoolchildren only noted their dietary habits related to the time in the lockdown. COVID-19 lockdown measures have been shown to pose a negative impact on an individual’s lifestyle habits, including decreased physical activity and an unhealthy diet, eventually resulting in weight gain [57], which we also demonstrated with this study’s results among school children. The observed lifestyle changes among children could lead to numerous pathophysiological disturbances if they persist into adulthood, such as the development of insulin resistance, diabetes, and many other diseases involving inflammatory pathways [57]. Recently, an increase in new-onset diabetes due to COVID-19 disease was shown, even in children [58] and, if this increase is joined by that resulting from lifestyle changes and obesity prevalence increase, we could witness a serious diabetes incidence increase.

## 5. Conclusions

In summary, data from this web-based survey showed an increase in overweight and obesity among Croatian schoolchildren during the COVID-19 lockdown. They changed their lifestyle habits toward being less physically active, spending more time using screen-based media and revealing potential psychological distress. However, observed healthy dietary habits could be a reflection of accepted messages of nutritional recommendations during the COVID-19 lockdown by their parents. Therefore, it is important to use the parental influence of supervision on their children about the importance of physical activity and healthy diet, as well as the responsible use of screen-based media, which are all important for mental and overall health. The study data are valuable for planning future public health programs that promote a healthy lifestyle in the school environment, aimed at both children and their parents, which could have an impact on the better health of school children providing them with a healthy adulthood.

## Figures and Tables

**Table 1:** Characteristics and lifestyle habits of participants according to gender before and during COVID-19 lockdown.

	Before COVID-19 Lockdown							During COVID-19 Lockdown								
Variables	Boys (*N* = 639)		Girls (*N* = 731)		Total (*N* = 1370)		*p*-Value ^a^	Boys (*N* = 639)		Girls (*N* = 731)		Total (*N* = 1370)		*p*-Value ^b^	Boys *p*-Value ^c^	Girls *p*-Value ^d^
BMI (kg/m^2^) *	20.53 ± 3.88		19.98 ± 3.84		20.23 ± 3.87		0.010 ^e^	21.22 ± 6.95		20.60 ± 3.69		20.89 ± 5.47		0.041 ^e^	0.029 ^f^	0.002 ^g^
BMI-for-age level	*N*	%	*N*	%	*N*	%		*N*	%	*N*	%	*N*	%			
Underweight	38	6.0	48	6.6	86	6.3	0.344	14	2.2	18	2.5	32	2.3	0.607	0.007	0.001
Normal weight	456	71.4	542	74.2	998	72.9		461	72.1	548	75.0	1009	73.7			
Overweight	104	16.3	94	12.9	198	14.5		117	18.3	118	16.1	235	17.2			
Obese	41	6.4	47	6.4	88	6.4		47	7.4	47	6.4	94	6.9			
Physical activity level	*N*	%	*N*	%	*N*	%		*N*	%	*N*	%	*N*	%			
Low	104	16.3	161	22.0	265	19.3	0.003	484	75.7	551	75.4	1035	75.6	0.954	<0.001	<0.001
Moderate	522	81.7	565	77.3	1087	79.3		149	23.3	174	23.8	323	23.6			
High	13	2.0	5	0.7	18	1.3		6	0.9	6	0.8	12	0.9			
MET-min/week *	3939.4 ± 894.9		3703.9 ± 812.2		3813.7 ± 859.2		<0.001 ^e^	2470.5 ± 386.6		2469.8 ± 337.9		2470.1 ± 180.3		0.472 ^e^	<0.001	<0.001
Organized activities (sports)	*N*	%	*N*	%	*N*	%		*N*	%	*N*	%	*N*	%			
<2 d/week	158	24.7	255	34.9	413	30.2	<0.001	165	25.8	256	35.0	421	30.7	<0.001	0.878	<0.001
2–3 d/week	251	39.3	247	33.8	498	36.4		251	39.3	354	48.4	605	44.2			
≥4 d/week	230	36.0	229	31.3	459	33.5		223	34.9	121	16.6	344	25.1			
Non-organized activities (games)	*N*	%	*N*	%	*N*	%		*N*	%	*N*	%	*N*	%			
<2 d/week	99	15.5	150	20.5	249	18.2	0.005	142	22.2	222	30.4	364	26.6	0.002	0.007	<0.001
2–3 d/week	208	32.6	262	35.8	470	34.3		181	28.3	202	27.6	383	28.0			
≥4 d/week	332	52.0	319	43.6	651	47.5		316	49.5	307	42.0	623	45.5			
Class/homework sitting	*N*	%	*N*	%	*N*	%		*N*	%	*N*	%	*N*	%			
>7 h/day	375	58.7	487	66.6	862	62.9	0.002	459	71.8	476	65.1	935	68.3	0.008	<0.001	0.544
<7 h/day	264	41.3	244	33.4	508	37.1		180	28.2	255	34.9	435	31.8			
PC/tablet/mobile phone use	*N*	%	*N*	%	*N*	%		*N*	%	*N*	%	*N*	%			
<2 h/day	178	27.9	204	27.9	382	27.9	0.964	113	17.7	115	15.7	228	16.6	0.333	<0.001	<0.001
≥2 h/day	461	72.1	527	72.1	988	72.1		526	82.3	616	84.3	1142	83.4			
TV watching	*N*	%	*N*	%	*N*	%		*N*	%	*N*	%	*N*	%			
<2 h/day	588	92.0	677	92.6	1265	92.3	0.680	555	86.9	629	86.1	1184	86.4	0.663	0.003	<0.001
≥2 h/day	51	8.0	54	7.4	105	7.7		84	13.2	102	14.0	186	13.6			
Sleeping (h/day) *	7.83 ± 0.95		7.73 ± 0.94		7.77 ± 0.95		0.067 ^e^	8.09 ± 0.73		8.04 ± 0.71		8.06 ± 0.72		0.159 ^e^	<0.001 ^f^	<0.001 ^g^
	*N*	%	*N*	%	*N*	%		*N*	%	*N*	%	*N*	%			
≤7 h/day	378	59.2	461	63.1	839	61.2	0.319	357	55.9	451	61.7	808	59.0	0.032	0.001	0.044
8–9 h/day	12	1.9	11	1.5	23	1.7		37	5.8	26	3.6	63	4.6			
>9 h/day	249	39.0	259	35.4	508	37.1		245	38.3	254	34.8	499	36.4			

* Values are presented as means and SD; ^a^ Chi-squared test for differences between boys and girls before on-line (*p* < 0.05); ^b^ Chi-squared test for differences between boys and girls during on-line (*p* < 0.05); ^c^ McNemar’s test for differences between boys before and during on-line (*p* < 0.05); ^d^ McNemar’s test for differences between girls before and during on-line (*p* < 0.05); ^e^ Student’s *t*-test between boys and girls (*p* < 0.05); ^f^ Student’s *t*-test between boys before and during on-line (*p* < 0.05); ^g^ Student’s *t*-test between girls before and during on-line (*p* < 0.05).

**Table 2:** Dietary habits and nutrition knowledge of participants during COVID-19 lockdown.

	During COVID-19 Lockdown						
Variables	Boys (*N* = 639)		Girls (*N* = 731)		Total (*N* = 1370)		*p*-Value ^a,b^
KIDMED score (points) *	9.81 ± 2.13		9.70 ± 2.15		9.75 ± 2.14		0.311 ^a^
KIDMED tertiles	*N*	%	*N*	%	*N*	%	
low	2	0.3	5	0.7	7	0.5	0.547 ^b^
moderate	90	14.1	110	15.1	200	14.6	
high	547	85.6	616	84.3	1163	84.9	
Nutrition knowledge score (points) *	8.83 ± 1.94		9.17 ± 1.89		9.02 ± 1.92		0.001 ^a^
Nutrition knowledge tertiles	*N*	%	*N*	%	*N*	%	
low	25	3.9	18	2.5	43	3.1	0.007 ^b^
moderate	202	31.6	185	25.3	387	28.3	
high	412	64.5	528	72.2	941	68.7	

* Values are presented as means and SD; ^a^ Student’s *t*-test between boys and girls (*p* < 0.05); ^b^ Chi-squared test for differences between boys and girls (*p* < 0.05).

**Table 3:** Compliance with KIDMED score items among schoolchildren (*N* = 1370) according to gender, physical activity level and BMI-for-age classes (%).

KIDMED Score	Boys (*N* = 639)	Girls (*N* = 731)	*p*-Value *	Non-Active (*N* = 1035)	Active (*N* = 335)	*p*-Value *	Underweight (*N* = 32)	Normal Weight (*N* = 1009)	Overweight (*N* = 235)	Obesity (*N* = 94)	*p*-Value *
a fruit/day	72.6	73.6	0.682	73.4	72.2	0.669	75.0	73.7	73.6	64.9	0.318
second fruit/day	53.1	53.8	0.793	51.9	57.0	0.102	53.1	54.5	51.9	41.5	0.110
vegetables/day	100.0	100.0	0.999	100.0	100.0	0.999	100.0	100.0	100.0	100.0	0.999
more vegetable/day	50.8	55.6	0.075	37.7	41.2	0.251	40.0	53.9	45.6	58.6	0.038
fish ≥ 2 times/week	35.8	30.4	0.032	33.3	31.6	0.567	37.5	34.2	31.1	22.3	0.103
legumes ≥ 2 times/week	67.9	59.8	0.002	62.9	65.7	0.359	50.0	62.7	66.8	69.2	0.159
grain products ≥ 5 times/week	46.0	40.5	0.037	42.9	43.6	0.827	50.0	43.5	41.3	40.4	0.733
whole grain cereals and products for breakfast	80.9	82.4	0.490	82.0	80.6	0.556	84.4	82.9	78.3	76.6	0.212
milk, dairy for breakfast	87.0	83.2	0.047	84.3	87.2	0.195	87.5	85.6	84.9	78.7	0.334
no baked goods or pastries for breakfast	53.1	56.5	0.201	53.7	58.5	0.126	40.6	53.3	60.9	61.7	0.033
nuts ≥ 2 times/week	59.0	63.9	0.064	61.4	62.4	0.735	65.6	62.2	60.9	55.3	0.569
yogurt or cheese every day	75.6	69.5	0.012	71.9	73.7	0.511	78.1	75.0	70.6	67.0	0.209
no sweets or candies every day	58.5	50.2	0.002	53.2	56.7	0.267	56.3	51.6	60.4	63.8	0.019
fast food ≤ 1 times/week	78.1	83.6	0.010	80.3	83.3	0.225	90.6	80.7	83.0	76.6	0.286
olive oil every day	77.9	82.2	0.047	79.4	82.7	0.192	81.3	81.2	78.7	73.4	0.298
do not skip breakfast	84.0	77.0	0.001	91.3	93.4	0.217	93.8	92.9	88.5	88.3	0.086

* Chi-squared test for differences between boys and girls, non-active and active children, and between underweight, normal weight, overweight and obesity group (*p* < 0.05).

**Table 4:** Association of participants’ characteristics and lifestyle habits to overweight and obesity before and during COVID-19 lockdown.

Predictors	Outcome—Overweight and Obesity before COVID-19 Lockdown (*N* = 286)						Outcome—Overweight and Obesity during COVID-19 Lockdown (*N* = 329)					
	*N*	(%)	OR	95% CI		*p*-Value	*N*	(%)	OR	95% CI		*p*-Value
Gender												
Boys	146	51.1	1				164	49.9	1			
Girls	141	49.3	0.81	0.63	1.06	0.061	165	50.2	0.84	0.66	1.08	0.091
Age (categories)												
10–11 years	58	20.3	1				69	21.0	1			
12–13 years	166	58.0	0.99	0.70	1.39	0.471	191	58.1	0.94	0.68	1.30	0.359
14–15 years	63	22.0	0.58	0.39	0.87	0.004	69	21.0	0.51	0.35	0.75	0.001
Residence												
Rural	78	27.3	1				96	29.2	1			
Urban	208	72.7	1.07	0.80	1.43	0.329	233	70.8	0.95	0.72	1.24	0.346
Physical activity level												
Low	68	23.8	1				251	76.3	1			
Moderate	214	74.8	0.71	0.52	0.97	0.016	75	22.8	0.94	0.70	1.27	0.352
High	3	1.1	0.58	0.16	2.06	0.199	3	0.9	1.04	0.28	3.88	0.476
Organized activities (sports)												
<2 d/week	104	36.4	1				174	52.9	1			
2–3 d/week	83	29.0	0.59	0.43	0.82	0.001	85	25.8	0.23	0.17	0.31	<0.001
≥4 d/week	99	34.6	0.82	0.60	1.12	0.104	70	21.3	0.36	0.26	0.50	<0.001
Non-organized activities (games)												
<2 d/week	70	24.5	1				100	30.4	1			
2–3 d/week	87	30.4	0.58	0.41	0.83	0.002	87	26.4	0.78	0.56	1.08	0.067
≥4 d/week	129	45.1	0.63	0.45	0.89	0.004	142	43.2	0.78	0.58	1.05	0.050
Sedentary activities												
Class sitting/homework												
≥7 h/day	186	65.0	1				234	71.1	1			
<7 h/day	100	35.0	0.89	0.68	1.17	0.203	95	28.9	1.19	0.91	1.57	0.099
PC/tablet/mobile phone use												
<2 h/day	88	30.8	1				58	17.6	1			
≥2 h/day	198	69.2	0.83	0.63	1.11	0.111	271	82.4	0.94	0.68	1.31	0.366
TV watching												
≥2 h/day	24	8.4	1				51	15.5	1			
<2 h/day	262	91.6	1.13	0.71	1.83	0.302	278	84.5	1.23	0.87	1.75	0.121
Sleeping												
≤7 h/day	191	66.8	1				216	65.7	1			
8–9 h/day	3	1.1	0.51	0.15	1.73	0.140	12	3.7	0.65	0.34	1.23	0.092
>9 h/day	92	32.2	0.75	0.57	0.99	0.021	101	30.7	0.93	0.71	1.23	0.308

**Table 5:** Association of participants’ dietary habits and nutrition knowledge to overweight and obesity during COVID-19 lockdown.

Predictors	Outcome—Overweight and Obesity during COVID-19 Lockdown (*N* = 329)					
	*N*	%	OR	95% CI		*p*-Value
KIDMED						
Low	2	0.6	1			
Moderate	47	14.3	0.77	0.14	4.09	0.379
High	280	85.1	0.79	0.15	4.11	0.391
Breakfast						
<1 day/week	38	11.6	1			
1–3 day/week	46	14.0	0.72	0.43	1.20	0.103
4–6 day/week	75	22.8	0.46	0.28	0.73	0.001
Every day	170	51.7	0.63	0.41	0.97	0.019
Fruits						
<1 piece/day	168	51.1	1			
≥1 piece/day	161	48.9	4.42	3.39	5.76	<0.001
Vegetables						
1 meal/day	195	59.3	1			
≥2 meal/day	134	40.7	2.04	1.60	2.62	<0.001
Sweetened beverages						
≥1 drink/day	14	4.3	1			
<1 drink/day	184	55.9	8.64	6.34	11.77	<0.001
Fast food						
≥1 meal/day	62	18.8	1			
<1 meal/day	267	81.2	0.86	0.62	1.18	0.176
Sweet/salty snacks						
≥1/day	127	38.6	1			
1/day	202	61.4	1.48	1.15	1.91	0.001
Nutrition knowledge level						
Low	9	2.7	1			
Moderate	114	34.7	12.95	6.44	26.04	<0.001
High	206	62.6	8.69	4.40	17.18	<0.001

## Data Availability

The data presented in this study are available on reasonable request from the corresponding author. The data are not publicly available due to restrictions e.g., their containing information that could compromise the privacy of research participants.

## Supplementary Materials

The following are available online at https://www.mdpi.com/article/10.3390/nu13113788/s1, Table S1. Correctly answered nutrition knowledge score items among schoolchildren according to gender (%); Table S2. Schoolchildren’ feelings and worries about COVID-19 lockdown and staying at home (%).
