# Supplementary material for: The Outcome of COVID-19 Lockdown on Changes in Body Mass Index and Lifestyle among Croatian Schoolchildren: A Cross-Sectional Study
Source: Nutrients. 2021 Oct 26;13(11):3788. doi: 10.3390/nu13113788 (PMC8624838; doi:10.3390/nu13113788)
Supplement: Supplementary file 1 [file nutrients-13-03788-s001.zip › nutrients-1408685-supplementary.pdf]

Table S1. Correctly answered nutrition knowledge score items among schoolchildren (N=1370) according to gender (%).

Question	Boys (N=639)	Girls (N=731)	p-value*
How many pieces of fruit a child of your age should eat every day?	88.3	89.5	0.825
How many daily meals of vegetables a child of your age should eat every day?	62.8	69.4	0.370
Mark what you consider as a healthy breakfast.	87.6	93.2	0.228
Mark what you consider as a healthy meal.	95.2	96.7	0.470
Which of the following food group should you eat the most in a day?	90.3	93.4	0.447
Which food group should you eat the least?	83.4	90.2	0.147
Which food group is a good source of vitamin C?	75.3	79.9	0.397
Which food group is a good source of energy?	50.9	48.6	0.777
Which food group is a good source of calcium?	59.8	62.1	0.772
Which food group provides the most protein?	75.3	73.6	0.747
Which food group is a good source of fibers?	12.1	14.4	0.674
Which of the following nutrients gives the most energy?	23.9	24.4	0.962
How much water should we drink each day?	87.0	92.5	0.157
How much should children be physically active each day?	73.1	66.8	0.355

*Chi-squared test for differences between boys and girls ($p < 0.05$).

Table S2. Schoolchildren' feelings and worries about COVID-19 lockdown and staying at home (%).

	Boys (N=639)			Girls (N=731)			Total (N=1370)			
	No; not at al.	Neither no, neither yes.	Yes; definitely yes.	No; not at al.	Neither no, neither yes.	Yes; definitely yes.	No; not at al.	Neither no, neither yes.	Yes; definitely yes.	p-value
I feel lonelier.	57.6	23.3	19.1	47.3	32.8	19.8	52.1	28.3	19.5	<0.001
I feel happier.	37.4	32.1	30.5	41.6	31.2	27.2	39.5	31.7	28.8	<0.001
I miss my school friends.	16.6	18.8	64.6	18.1	21.3	60.6	17.3	20.2	62.5	<0.001
I feel more successful in my learning.	38.2	31.3	30.7	39.4	30.4	30.2	38.8	30.8	30.4	<0.001
I'm afraid to go back to school, I rather stay at home.	55.1	19.9	25.0	45.1	25.0	29.8	49.9	22.6	27.6	<0.001

*Chi-squared test for differences between boys and girls ($p < 0.05$).
